# The Microscopist

**Published:** 1851-10

**Authors:** 


*The* Microscopist, *or a Complete Manual on the Use of the Microscope, for Physicians, Students, and all Lovers of Natural Science, with illustrations.* By Joseph H. Wythers, M. D.

The above is the title of a small volume, comprising, in a small compass, all that is interesting and useful to be known upon the subject which it treats.

The first chapter is devoted to the history and importance of microscopic investigations. The second and third, to the microscope itself, and its different varieties with their several adjuncts.

The fourth and fifth teach the method of using the instrument with the manner of mounting and preserving objects for examination. Sixth and seventh treat where objects for examination are to be procured, and what is called "test objects." The eighth shows the manner of dissecting objects for the microscope—and the ninth, tenth, eleventh, twelfth and thirteenth chapters are devoted to the cell doctrine, morbid structures, minute injections, examination of urinary deposits, and polarized light.

